# 米托蒽醌脂质体联合化疗方案治疗混合表型急性白血病的安全性及有效性研究

**DOI:** 10.3760/cma.j.cn121090-20241210-00554

**Published:** 2025-01

**Authors:** 慧雯 江, 聪 卢, 静 何, 求哲 魏, 梅芳 苏, 耀辉 吴, 俊斌 胡

**Affiliations:** 1 华中科技大学同济医学院附属协和医院血液病研究所，武汉 430022 Institute of Hematology, Union Hospital, Tongji Medical College, Huazhong University of Science and Technology, Wuhan 430022, China; 2 黄冈市中心医院血液科，黄冈 438021 Department of Hematology, Huanggang Central Hospital, Huanggang 438021, China

**Keywords:** 米托蒽醌脂质体, 混合表型急性白血病, 有效性, 安全性, Mitoxantrone liposome, Mixed phenotype acute leukemia, Effectiveness, Safety

## Abstract

**目的:**

分析米托蒽醌脂质体（MIT-LIP）联合化疗方案治疗混合表型急性白血病（MPAL）的安全性及有效性。

**方法:**

回顾性纳入2021年12月至2024年11月于华中科技大学同济医学院附属协和医院和黄冈市中心医院接受过MAED（MIT-LIP+阿糖胞苷+依托泊苷+地塞米松）方案的MPAL患者，收集患者的临床特点、不良反应、治疗效果、长期预后等数据。

**结果:**

共纳入7例接受过MAED方案化疗的MPAL患者，其中2例初始诊断分别为急性T淋巴细胞白血病和急性B淋巴细胞白血病，经过治疗后表型转换为急性髓系白血病，3例初始诊断为髓/B双克隆MPAL，1例初始诊断为髓/T双克隆MPAL，1例初始诊断为髓/浆细胞样树突状细胞双克隆MPAL。7例患者中，男3例，女4例；中位年龄38（16～58）岁；检测到染色体异常1例，基因异常6例，其中伴有BCR∷ABL融合基因1例。MAED方案化疗期间未发生明确相关的药物过敏及器官毒性，主要不良反应为血液系统毒性。经诱导缓解治疗后，所有患者均达到完全缓解（CR），2例患者仍处于巩固治疗中并维持微小残留病（MRD）阴性CR；1例患者维持MRD阳性CR；4例患者行异基因造血干细胞移植，2例维持MRD阴性CR，2例复发。目前中位随访时间为12个月，总生存（OS）率为100％，无复发生存（RFS）率为60％，中位OS时间和中位RFS时间未达到。

**结论:**

MAED方案治疗MPAL安全性良好，CR率高。

## Abstract (en)

**Objective:**

To evaluate the safety and efficacy of mitoxantrone liposome（MIT-LIP）combined chemotherapy in treating mixed phenotype acute leukemia（MPAL）.

**Methods:**

December 2021 to November 2024, MPAL patients who underwent the MAED（MIT-LIP + cytarabine + etoposide + dexamethasone）regimen were retrospectively analyzed. Data on clinical characteristics, adverse reactions, therapeutic outcomes, and long-term prognoses were collected.

**Results:**

A total of 7 MPAL patients who received MAED regimen were admitted. Among them, two patients were initially diagnosed with T-ALL or B-ALL, respectively, and transformed into AML after treatment. Three patients were initially diagnosed as MPAL（B/myeloid）, one as MPAL（T/myeloid）, and one with MPAL（myeloid/plasmacytoid dendritic cell）. Among the 7 patients, there were 3 males and 4 females, 1 chromosome abnormalities and 6 gene abnormalities, including 1 case with BCR∷ABL fusion gene. The median age was 38 years（range: 16–58 years）. There was no clear related drug allergy and organ toxicity during MAED regimen, and the main adverse effect was hematological toxicity. After induced chemotherapy, all patients achieved complete remission（CR）, 2 maintained MRD-negative CR and 1 maintained MRD-positive CR. The other 4 patients underwent allogeneic hematopoietic stem cell transplantation, 2 maintained MRD-negative CR, and 2 relapsed. The current median follow-up time was 12 months, the overall survival（OS）rate was 100％, the relapse-free survival（RFS）rate was 60％, and the median OS time and median RFS time were not reached.

**Conclusion:**

The MAED regimen demonstrates high safety and a favorable CR rate in MPAL treatment.

混合表型急性白血病（Mixed phenotype acute leukemia, MPAL）是一种高度异质性的血液系统恶性肿瘤，仅占急性白血病病例的2％～5％[1]。MPAL的诊断基于欧洲白血病免疫分型协作组（EGIL）与WHO发布的疾病分类及诊断标准。MPAL的特点在于白血病细胞同时表达髓系和淋系的抗原标志，存在双表型MPAL、双细胞系MPAL及细胞系转化的MPAL共3种形式。目前尚无针对MPAL患者的治疗指南，治疗手段主要包括化疗及异基因造血干细胞移植（allo-HSCT）[2]–[3]。一项纳入100例MPAL患者的临床研究表明，急性淋巴细胞白血病（ALL）方案诱导治疗的应答率为85％，急性髓系白血病（AML）方案的应答率为41％，ALL+AML联合方案的应答率为60％，5年总生存（OS）率为37％[4]。总体而言，MPAL治疗难度大，预后较差，亟需寻找更有效的治疗方案。基于临床经验，我们在MPAL患者的诱导及巩固治疗中创新性应用含米托蒽醌脂质体（Mitoxantrone liposome, MIT-LIP）的联合化疗方案，取得了较好疗效，现回顾性收集患者临床资料，以综合评估该方案的安全性和有效性。

## 病例与方法

1. 研究对象：本研究为回顾性临床研究，收集2021年12月至2024年11月接受过MAED（MIT-LIP+阿糖胞苷+依托泊苷+地塞米松）±维奈克拉联合化疗方案的MPAL患者资料，其中华中科技大学同济医学院附属协和医院5例、黄冈市中心医院2例。

2. 形态学分析：新鲜骨髓涂片经瑞氏-吉姆萨染色后在显微镜下观察细胞形态及分类情况，根据镜检结果酌情加做细胞化学染色。

3. 免疫学分型：患者骨髓常规标记抗体，经PBS洗涤后上机检测，标记抗体包括HLA-DR、CD1a、CD2、CD3、CD4、CD5、CD7、CD8、CD10、CD11b、CD13、CD14、CD15、CD16、CD19、CD20、CD22、CD33、CD34、CD38、CD56、CD64、CD71、CD99、CD117、CD123、MPO、cCD79a、cCD3、TdT、CD45。

根据2016年发布的WHO造血和淋巴组织肿瘤分类标准，髓系指MPO（流式细胞分析、免疫组织化学、细胞化学）或单核细胞分化标志物（至少包括两种相关标志物：非特异性酯酶细胞化学、CD11c、CD14、CD64、溶菌酶）阳性，T淋巴系指细胞质CD3（CD3ε链的抗体）或膜表面CD3强阳性，B淋巴系指CD19强阳性伴至少一项标志物强表达（CD79a、细胞质CD22或CD10）或CD19弱表达伴至少2项标志物强表达（CD79a、细胞质CD22或CD10）[5]。

4. 染色体核型分析：采用培养24 h后的骨髓细胞，应用G带（显带水平200～300）分析染色体核型，根据《人类细胞遗传学国际命名体制（ISCN2013）》描述异常核型。

5. 分子生物学检查：基于Illumina高通量测序系统，通过捕获探针法进行血液病基因二代测序，检测包括ABL1、CEBPA、DNMT3A、FLT3等在内的62个基因。

6. 治疗方案：患者均使用过MAED方案作为诱导缓解治疗或巩固治疗，方案为：MIT-LIP 20 mg/m^2^，静脉滴注，第1天；阿糖胞苷200 mg，静脉滴注，第1～5天；依托泊苷80 mg/m^2^，静脉滴注，第4～5天；地塞米松15 mg，静脉滴注，第1～5天。第1周期未缓解（NR）的患者，可加用维奈克拉。伴BCR∷ABL融合基因阳性的患者，可加用酪氨酸激酶抑制剂（TKI）。

7. 随访：采用电话或查阅病历的方式进行随访，随访时间截至2024年11月。生存随访的起始时间为初次诊断时间，OS时间的终点为死亡或末次随访时间，无复发生存（RFS）时间的终点为复发或末次随访时间。

8. 统计学处理：采用SPSS 29.0软件进行数据分析，计数资料以例数表示，计量资料以中位数（范围）表示，生存分析采用Kaplan-Meier法。

## 结果

1. 临床特征：2021年12月至2024年11月，共7例MPAL患者接受过MAED方案化疗，其中男3例，女4例；中位年龄38（16～58）岁。初诊时患者外周血中位WBC 57.32（2.49～245.10）×10^9^/L、HGB 74（59～111）g/L、PLT 52（23～527）×10^9^/L。具体临床特征见表1。

**表1:** 7例混合表型急性白血病患者初诊时临床特征

例号	性别	年龄（岁）	WBC（×10^9^/L）	HGB（g/L）	PLT（×10^9^/L）	骨髓增生是否活跃	骨髓原始细胞比例（％）
1	男	58	87.54	59	64	是	90.0
2	男	16	57.32	77	52	是	82.0
3	女	38	61.71	60	32	是	65.5
4	女	27	2.49	111	53	是	63.5
5	女	33	245.10	75	527	是	60.5
6	女	49	14.56	74	23	是	65.6
7	男	54	2.63	63	50	是	72.8

2. 形态学检查：初诊时所有患者均表现为骨髓增生活跃或极度活跃，中位骨髓原始细胞比例为65.6％（60.5％～90.0％）（表1）。根据FAB分型，7例MPAL患者呈现出形态学异质性，其中3例患者表现为ALL，3例患者表现为MPAL，1例患者表现为谱系不明的急性白血病。

3. 免疫分型：根据流式细胞术免疫分型结果，2例MPAL患者发生系别转换，初始诊断分别为T-ALL和B-ALL，经过治疗后转换为AML；另外5例MPAL患者为双克隆，其中3例髓/B双克隆，1例髓/T双克隆，1例髓/浆细胞样树突状细胞双克隆。在所有患者中，髓系抗原阳性率由高到低依次为CD33（6例）、CD13（4例）、CD117（4例）、MPO（1例）、CD15（1例）；B系抗原阳性率由高到低依次为CD22（4例）、CD19（3例）、CD10（2例）、CD79a（2例）、CD20（1例）、CD24（1例）；T系抗原阳性率由高到低依次为CD7（4例）、CD5（3例）、CD2（2例）、cCD3（2例）、CD10（1例）。具体见表2。

**表2:** 7例混合表型急性白血病（MPAL）患者免疫学、细胞遗传学及分子生物学特征

例号	流式免疫分型	染色体	二代测序	诊断
1	初诊：92.3％为异常T系原始细胞；复查：89.97％为异常髓系原始细胞	正常	PHF6、JAK1、JAK3、NOTCH1	初始为T-ALL，后修正为MPAL（髓/T双克隆）
2	初诊：76.67％为异常B系原始细胞；复查：8.58％为异常髓系原始细胞	正常	未见异常	初始为B-ALL，后修正为MPAL（髓/B双克隆）
3	可见两群异常细胞，分别表达髓系及B系标志，考虑MPAL（髓/B）	正常	RUNX1	MPAL（髓/B双克隆）
4	47.63％为表型异常髓系原始细胞，伴部分B系标志表达（部分弱表达cCD79a和CD22）；5.99％为表型异常T系原始细胞，考虑MPAL（髓/T双克隆），建议密切随访髓/T/B三系标志	未做	SETD2、KMT2C、KRAS、BCOR	MPAL（髓/T双克隆）
5	34.20％为表型异常的B系原始细胞，伴部分髓系标志（CD13，CD33）及部分T系标志（CD7，CD5^dim^）表达，另见1.28％为表型异常的髓系原始细胞，伴部分B系标志表达（CD19，cCD79a^dim^）；考虑MPAL（B/髓双克隆）可能性大，建议密切随访B/髓两系标志	46,XX,t（9;22）[10]/46,idem,del（2）（q11q23）[5]/47,idem,+21[5]	BCR::ABL、RUNX1、ASXL1	MPAL（髓/B双克隆）
6	约81.5％表达HLA-DR、CD19、CD33、CD34、CD38、CD123、cCD22（dim），少量表达CD36，考虑MPAL（B/髓双克隆）可能性大	正常	ETV6、FLT3、IDH1、CSMD1、IKZF1、LMO2、LYL1、LCF7L2	MPAL（髓/B双克隆）
7	原始细胞分布区域可见异常细胞群体，约占51.5％；其中阳性表达CD5^dim^、CD7^bri^、CD11b、CD33、CD34、cD99、CD38、CD58、TdT。髓系区域约占33.5％，其中可见约24％的CD123^bri+^细胞，同时表达HLA-DR、CD2、CD4、CD11b、CD33、CD36、CD303、CD304，考虑为浆细胞样树突状细胞，其余约9.5％为髓系细胞，提示为急性白血病，系别分类不明，浆细胞样树突状细胞比例明显增高	正常	NUP98::HOXA9	MPAL（髓/浆细胞样树突状细胞双克隆）

**注** ALL：急性淋巴细胞白血病

4. 细胞遗传学及分子生物学特征：7例患者中有6例进行染色体检测，其中1例染色体异常，表现为46,XX,t（9;22）[10]/46,idem,del（2）（q11q23）[5]/47,idem,+21[5]。7例患者全部进行血液病基因二代测序，6例伴有异常突变基因，其中1例伴有BCR∷ABL p210型阳性。另检测出19种已知突变基因，包括RUNX1、PHF6、FLT3、IDH1、KMT2C、NOTCH1等（表2）。

5. 不良反应：随访期间，MAED方案化疗共实施18个周期（表3），患者未发生与MIT-LIP明确相关的药物过敏及心脏、肝脏、肾脏等器官毒性。化疗相关主要不良反应为血液系统毒性，其中粒细胞缺乏发生率为100％，中位持续时间为7.5 d；Ⅳ度血小板减少发生率为83.3％，Ⅲ度血小板减少发生率为16.7％。

**表3:** 7例混合表型急性白血病患者治疗经过及结局

例号	MAED诱导治疗周期数	MAED巩固治疗周期数	是否行allo-HSCT	疾病状态^a^
1	1	1	否	MRD^+^ CR
2	1	1	是	MRD^-^ CR
3	1	2	是	MRD^+^ CR
4	1	3	是	MRD^-^ CR
5	2	0	是	复发
6	1	1	否	MRD^-^ CR
7	0	3	否	MRD^-^ CR

**注** MAED：米托蒽醌脂质体+阿糖胞苷+依托泊苷+地塞米松；allo-HSCT：异基因造血干细胞移植；MRD：微小残留病；CR：完全缓解。^a^末次随访时疾病状态

6. 治疗方案与疗效：2例初始诊断为ALL的患者均接受以VDP方案（长春新碱+柔红霉素+泼尼松）为基础的诱导化疗。例1在方案中加用培门冬酰胺酶、环磷酰胺及西达本胺，经历2个周期诱导化疗后出现疾病进展（PD），复查骨穿发现T系克隆消失而髓系克隆表达，故修正诊断为MPAL（髓/T双克隆），随后接受2个周期MEAD联合维奈克拉方案化疗，可维持微小残留病（MRD）阳性完全缓解（CR）。例2在诱导方案中加用环磷酰胺，1个周期化疗后取得MRD阴性CR，后因全血细胞减少、粒细胞缺乏并发热入院，复查骨穿提示复发，表达髓系克隆，故修正诊断为MPAL（髓/B双克隆），随后接受2个周期MEAD方案化疗、1个周期HyperCVAD courseB方案化疗，并顺利桥接allo-HSCT，持续MRD阴性CR。

例4初始诊断为MPAL（髓/T双克隆），在接受4个周期MAED方案化疗后行allo-HSCT，持续MRD阴性CR。例7初始诊断为MPAL（髓/浆细胞样树突状细胞双克隆），在2个周期DAE方案（柔红霉素+阿糖胞苷+依托泊苷）化疗后MRD仍未转阴，后更换为MAE方案（MIT-LIP+阿糖胞苷+依托泊苷）化疗，MRD顺利转阴。

3例MPAL（髓/B双克隆）患者中，例3接受包含MAED方案在内的4个周期化疗，化疗期间持续MRD阴性CR，桥接allo-HSCT后第51天复发，予以MAED方案化疗联合供者干细胞二次回输后，再次达到MRD阴性CR，后MRD转阳。例5伴有BCR∷ABL融合基因，接受1个周期MAED方案化疗联合奥雷巴替尼治疗，疾病部分缓解（PR）且基因未完全转阴，在第2周期治疗中加用维奈克拉，取得MRD阴性CR且融合基因转阴，第3周期行HyperCVAD courseB+奥雷巴替尼+维奈克拉治疗，MRD及融合基因又转阳，桥接allo-HSCT后MRD及融合基因再次转阴，但在移植后第118天疾病复发。例6接受HyperCVAD courseA+阿糖胞苷+维奈克拉联合化疗1个周期后达到PR，后续更换为MAED联合维奈克拉方案化疗，2个周期后达到MRD阴性CR。

截至2024年11月，4例患者处于MRD阴性CR状态，2例患者处于MRD阳性CR状态，1例患者处于复发状态（表3）。目前中位随访时间为12个月，OS率为100％，RFS率为60％，中位OS时间及RFS时间未达到（图1）。

**图1:**
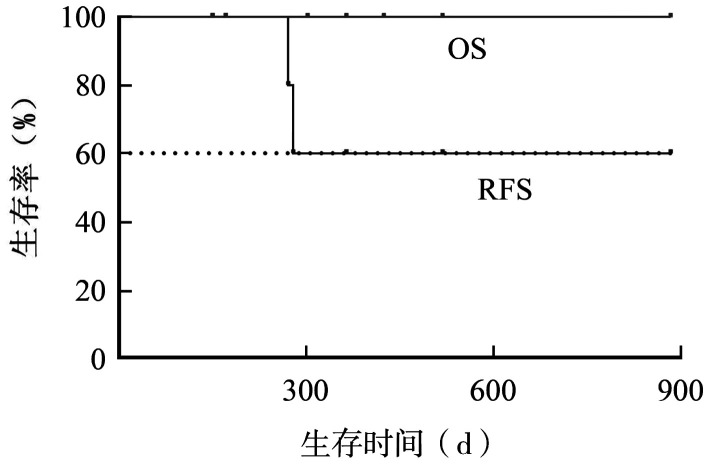
7例混合表型急性白血病患者总生存（OS）及无复发生存（RFS）曲线

## 讨论

MPAL是一类复杂且异质性高的急性白血病，细胞形态学上可能表现为ALL、AML、MPAL或无法分类等[4]。本研究中，3例患者在形态学上表现为ALL，3例表现为MPAL，1例谱系不明，通过流式细胞术、免疫组化等手段进一步识别白血病细胞的分化特征后，明确诊断为MPAL。2016年发布的WHO标准强调，若MPAL白血病细胞明确存在2种及以上克隆细胞亚群时，无需严格遵从WHO2008标准，只需各细胞亚群分别满足髓系或T/B细胞系白血病的诊断，即可诊断为MPAL[5]。除双表型、双细胞系MPAL以外，还存在细胞系转化的MPAL。本研究中有2例患者初始诊断为ALL，复发后细胞系转化为AML。回顾初诊时免疫分型结果，发现例1原始细胞群具有CD13、CD117等髓系分化特征，不排除该患者在初诊时即为MPAL，诱导化疗抑制了占主要优势的白血病克隆，而另一种不同表型的亚克隆大量扩增，表现为细胞系转化。另外，2例患者初诊时的原始细胞群均表达非谱系特异性抗原CD34，提示该群细胞有向淋系或髓系分化的潜能，最近的单细胞测序分析也发现，MPAL细胞表达共同的干细胞样转录谱，具有高分化潜力[6]。考虑以上因素，2例患者均修正诊断为MPAL。

MPAL患者的生物学特征各异，目前尚无针对MPAL的诊疗指南，既往临床经验提示ALL方案优于AML方案，且推荐患者行allo-HSCT[7]–[9]。近期一项纳入77例患者的多中心回顾性临床研究中，30例（39.0％）患者接受基于氟达拉滨的AML样诱导化疗，47例（61.0％）接受以儿科方案为主的ALL样诱导化疗，71例可评估疗效。48例（67.6％）患者达到CR，其中20例（41.7％）为MRD阴性，且ALL样治疗与更好的CR率相关[10]。近两年也有一些新方案在MPAL患者中应用。在纪念斯隆凯特琳癌症中心，17例MPAL患者接受ALL-2方案（高剂量阿糖胞苷+米托蒽醌）治疗的总有效率为94％[11]。另一项临床研究提示，MPAL患者接受CLAG-M方案（克拉屈滨+阿糖胞苷+G-CSF+米托蒽醌）作为诱导缓解或巩固治疗后75％（12/16）可获得PR或CR[12]。米托蒽醌是一种DNA合成抑制剂，通过干扰细胞的增殖周期，发挥抗肿瘤作用。脂质体作为载体，能够将米托蒽醌包裹在内，从而提高药物的稳定性和生物利用度。MIT-LIP通过静脉注射进入体内后，能够迅速被肿瘤细胞摄取，并释放出米托蒽醌，发挥其抗肿瘤活性。本研究中将MIT-LIP与阿糖胞苷、依托泊苷、地塞米松联合使用，组成MAED化疗方案。MAED作为诱导缓解方案在6例患者中使用，其中3例为初始诱导治疗，另外3例为复发后的再诱导治疗，经诱导后5例患者达到MRD阴性CR，1例达到MRD阳性CR，CR率为100％。同时，MAED也可作为巩固化疗方案，在本研究中应用11个周期，其中10个周期疗效评估为MRD阴性CR，1个周期评估为MRD阳性CR。值得注意的是，例7为MPAL（髓/浆细胞样树突状细胞双克隆），使用含柔红霉素化疗方案2个疗程后，MRD仍为阳性，将柔红霉素更换为MIT-LIP后，予以MAED方案化疗，MRD顺利转阴，提示MIT-LIP的肿瘤浸润能力更强。总体而言，本研究中所采用MAED方案的总体有效率高于既往临床研究，目前我中心已通过伦理审查，未来将开展前瞻性临床试验以获取更多临床数据。

除常规化疗以外，靶向治疗可针对特定的癌细胞靶点，具有更强的针对性和更低的不良反应，从而增强MPAL的治疗效果。Ph染色体或BCR∷ABL融合基因阳性MPAL是一种单独亚型，具有发病率低、初诊时WBC高等特点，治疗上建议加用TKI[7],[13]–[14]。既往研究提示，BCR∷ABL阳性为MPAL的预后不良因素[4]。本研究中有1例BCR∷ABL阳性MPAL，在MAED诱导化疗基础上，加用第三代TKI奥雷巴替尼，经1个周期治疗后患者未达到CR，但BCR∷ABL融合基因定量有所下降。维奈克拉是一种BCL-2抑制剂，已有个例报道用于治疗MPAL，疗效良好[15]–[16]。第2周期诱导化疗中，在MAED联合奥雷巴替尼的基础上再加用维奈克拉，患者可达到MRD阴性CR，且BCR∷ABL融合基因转阴。患者随后接受1个周期HyperCVAD courseB+奥雷巴替尼+维奈克拉巩固治疗，复查骨穿发现MRD及融合基因转阳。尽管患者行allo-HSCT后MRD及融合基因再次转阴，但在移植后第118天疾病复发。既往研究指出，MPAL患者移植前的肿瘤负荷与移植后的预后相关[17]。对于MRD阳性MPAL患者，或可增加MAED巩固治疗周期数，尽量使患者MRD转阴后再桥接移植，降低移植后复发率。另有研究证实，对于移植后复发的MPAL患者，根据白血病系别特征采用靶向CD19或CD7的CAR-T细胞治疗可再次诱导缓解[18]–[20]；维奈克拉联合阿扎胞苷或达雷妥尤单抗也具有良好疗效[21]–[22]。

综上所述，MPAL的诊断需借助细胞形态学、免疫学、分子遗传学等多种检查手段，并建议及时复查骨穿，评估治疗中是否发生谱系变化。与既往研究相比，MAED化疗方案在MPAL治疗中具有良好的安全性和有效性，有望改善MPAL患者预后。
